# The effect of a worksite based walking programme on cardiovascular risk in previously sedentary civil servants [NCT00284479]

**DOI:** 10.1186/1471-2458-6-136

**Published:** 2006-05-22

**Authors:** Marie H Murphy, Elaine M Murtagh, Colin AG Boreham, Lesley G Hare, Alan M Nevill

**Affiliations:** 1 Sport and Exercise Sciences Research Institute, University of Ulster, Northern Ireland, UK; 2 Institute of Clinical Science, Department of Medicine, Queen's University, Belfast, Northern Ireland, UK; 3 Research Institute of Healthcare Sciences, University of Wolverhampton, England, UK

## Abstract

**Background:**

A significant proportion of Europeans do not meet the recommendations for 30 mins of physical activity 5 times per week. Whether lower frequency, moderate intensity exercise alters cardiovascular disease (CVD) risk has received little attention. This study examined the effects of 45 minutes self-paced walking, 2 d· wk^-1 ^on aerobic fitness, blood pressure (BP), body composition, lipids and C-Reactive Protein (CRP) in previously sedentary civil servants.

**Methods:**

37 subjects (24 women) aged 41.5 ± 9.3 years were randomly assigned to either two 45 minute walks per week (walking group) or no training (control group). Aerobic fitness, body composition, blood pressure (BP), CRP and lipoprotein variables were measured at baseline and following 8 weeks. Steps counts were measured at baseline and during weeks 4 and 8 of the intervention.

**Results:**

Compared to the control group, the walking group showed a significant reduction in systolic BP and maintained body fat levels (*P *< 0.05). There were no changes other risk factors. Subjects took significantly more steps on the days when prescribed walking was performed (9303 ± 2665) compared to rest days (5803 ± 2749; *P *< 0.001).

**Conclusion:**

These findings suggest that walking twice per week for 45 minutes at ~ 62% HR_max_, improves activity levels, reduces systolic BP and prevents an increase in body fat in previously sedentary adults. This walking prescription, however, failed to induce significant improvements in other markers of cardiovascular disease risk following eight weeks of training.

## Background

It has become increasingly clear that many of the chronic diseases we face today are associated fundamentally with the pervasive sedentariness of modern lifestyle. In Northern Ireland at least 2000 people die each year due to an inactive lifestyle and the avoidable cost of inactivity to the health service is estimated at £0.62 million each year [1]. Despite the proven benefits of regular exercise (Kohl, 2001) and published activity guidelines [2], the majority of westernised societies do not undertake enough physical activity to confer a health protective benefit. It is estimated that 66% of Europeans are physically active for less than 30 minutes daily [3]. Given the strong association between physical inactivity and cardiovascular disease[4], and the prevalence of inactivity in today's population, increasing levels of physical activity represents great potential for public health gain.

In the past decade, physical activity research has been characterised by the development of exercise prescriptions that are palatable to sedentary populations in the western world. First advocated by the American College of Sports Medicine [5] and subsequently adopted by numerous consensus panels [6,7] the recommendation that "every adult should accumulate 30 minutes of moderate intensity activity on most, preferably all, days of the week" is now widely accepted. However, compliance with these guidelines requires considerable commitment in terms of time spent exercising per week (≥ 150 minutes) and this may deter individuals from starting an exercise programme. Accordingly, randomised controlled trials are needed to evaluate the effects of smaller volumes of exercise on health.

Walking is eminently suited to population exercise prescription as it is easy to do, requires no special skills or facilities, and is achievable by virtually all age groups with little risk of injury [8]. There is some evidence that a training frequency of as low as two days per week may elicit improvements in cardiorespiratory fitness in the lower fitness categories [9]. To the authors' knowledge, only two studies have examined the effects of a twice-weekly training intervention using walking as the exercise mode. A twice-weekly, six-month progressive walking programme (45 minutes per session) improved indices of aerobic fitness, but failed to alter blood pressure (BP) in 60–70 year old women [10]. Similarly, walking 25 minutes, 2 d· wk^-1 ^for six months, improved fitness in 79–91 year old females [11]. However, the effect of a low frequency walking prescription on fitness in younger adults remains to be investigated. Furthermore, the effects of low frequency, moderate intensity training on other markers of cardiovascular disease (CVD) risk have received very little attention. There is increasing recognition that traditional risk factors, such as hypercholesterolemia, hypertension and obesity, do not fully account for the occurrence of CVD. For example, almost one-half of the 1.3 million individuals who develop myocardial infarction in the United States each year have either normal or only moderately increased cholesterol concentrations [12]. Recently, C-Reactive Protein (CRP) has emerged as a novel risk factor for CVD. It has a strong association with cardiovascular risk, which is consistent, dose-related and independent. This is the first study to examine the effects of walking, without dietary intervention, on CRP in previously sedentary adults.

The present study evaluates the efficacy of a workplace walking intervention on physical activity levels and cardiovascular risk factors. An examination of the effects of self-paced walking twice per week will reveal whether individuals can achieve health benefits from outdoor walking performed with minimal investment in time. The purpose of the present study was to examine the effects of 45 minutes walking, 2 d· wk^-1 ^on fitness, BP, body composition, lipids and CRP.

## Methods

### Study design

This study was a randomised controlled trial, with subjects assigned to a walking or control group. The Research Ethics Committee at Queen's University, Belfast approved the study. Measurements were made at baseline (pre-intervention) and following eight weeks (post-intervention).

### Subjects

Subjects were recruited from staff at the Northern Ireland Civil Service via internal email. All subjects completed a Health History questionnaire. Exclusionary criteria were a physically active lifestyle (defined as compliance with current physical activity guidelines (Pate, 1995), age > 65 years, resting BP > 159/99 mm Hg, total cholesterol > 6.2 mmol· L^-1^, fasting blood glucose > 7.0 mmol· L^-1^, body mass index (BMI) > 34.9 kg· m^-2^, current cigarette smokers, individuals with cardiovascular, pulmonary or metabolic disease, pain or discomfort in the chest, dizziness or heart murmur. In addition, individuals taking medication known to interfere with lipid metabolism, and females who were pregnant or planning to become pregnant in the following five months were excluded from taking part in the study. Thirty seven subjects (24 women) aged 41.5 ± 9.3 years were randomised to either a walking (n = 23; 16 women) or control group (n = 14; 8 women) on a 3 to 2 basis (3 walkers for every 2 controls). All subjects gave their written informed consent.

### Measures

Height and body mass were recorded using a stadiometer (Seca model 770, Vogel & Halke; Hamburg, Germany) and scales (Seca model 707 digital physicians scale; Vogel & Halke, Hamburg, Germany) respectively. BMI was calculated by dividing body mass (kg) by height (m^2^). Waist measurements were made at the level of the trunk where the girth is minimal, i.e. the location where there was a noticeable indentation of the trunk. If there was no noticeable indentation the tape was located at the umbilicus. Hip girth was the horizontal circumference at the broadest part of the lower body, usually at the level of the trochanters [13]. Body fat percentage was assessed using bioelectrical impedance analysis following recommended procedures [14].

Duplicate measurements of BP were taken two minutes apart using an automated device (Omron 705 CP; Omron Matsusaka Co. Ltd., Japan) after the subject had rested in a seated position for five minutes. The average of the readings was used. If the first two readings differed by more than 5 mm Hg, an additional reading was obtained and all three readings averaged.

To monitor cardiovascular adaptations to training, a submaximal, graded exercise test was conducted pre- and post-intervention. In the week prior to the treadmill test, subjects were familiarised with walking on the treadmill (Vision Fitness HRC T8600; Lake Mills, U.S.) at various speeds and slopes. Following a three minute warm-up, subjects were instructed to walk on the treadmill for four minutes at each of four gradients selected to elicit 40, 50, 60, and 70% of age-predicted (220 – age) maximum heart rate (HR_max_). They were encouraged to complete all four stages, but the test was terminated if heart rate (HR) reached 85% HR_max_. HR was measured continually by short-range telemetry. During the last 30 seconds of each test stage, a capillary sample of blood was obtained and immediately analysed for lactate using a Lactate Pro Test Meter (Arkray Inc.; Kyoto, Japan). Ratings of perceived exertion (RPE) using the Borg 15-grade scale (Borg, 1982) were obtained during the last minute of each test stage, in accordance with scripted instructions [14]

Whole blood glucose and total cholesterol were determined from a fresh sample of capillary blood using dry chemistry methods (Reflotron analyser, Boehinger Mannheim Ltd.; UK) for the purpose of initial screening criteria. Blood samples were obtained by venepuncture after a 10 hour fast and with subjects in a seated position. Subjects were instructed to refrain from physical activity on the previous day. Samples were separated, frozen at -20°C, and analysed within 8 months. Total cholesterol, HDL and triglycerides were determined by automated enzyme assay using a Cobas FARA bioanalyser (Roche Products Ltd; Herts, UK). Commercial enzyme assay kits were purchased from Randox Laboratories Ltd. (Crumlin, UK). The concentration of LDL cholesterol was calculated using the Friedewald formula [15]. CRP was measured using an automated high sensitivity immunoturbidimetric assay (Randox Laboratories Ltd; Crumlin, UK) on a Cobas FARA bioanalyser (Roche Products Ltd; Herts, UK). Where CRP levels were below detectable levels (0.5 mg.L^-1^) a value of 0.5 mg.L^-1 ^was recorded. All samples for each given assay were analysed on the same day in a single batch at the Institute of Clinical Science at Queen's University, Belfast.

A sample of whole blood was analysed within 12 hours for haematocrit and haemoglobin. Haematocrit was corrected by 1.5% to account for plasma trapped between erythrocytes. Haematocrit and haemoglobin values were subsequently used to correct for changes in plasma volume [16]. As female hormonal changes throughout the menstrual cycle may influence lipid profiles [17], pre- and post-intervention samples were taken during the same phase of the menstrual cycle. In order that lipid parameters were not influenced by the acute effects of previous exercise [18], the post-intervention sample was obtained on day 3 after the last walking session.

### Walking programme

It has previously been reported that adults intuitively self-select a pace concordant with cardiorespiratory benefits when walking for exercise [19]. Therefore, in the present study, subjects were allowed to choose their own walking speed. The progressive walking programme lasted eight weeks. During week one, subjects completed a 25 minute walk on two days. During week two, subjects walked for 35 minutes on two days. From week three to week eight, all walkers completed two 45 minute walks per week. All walking sessions were performed outdoors. Those assigned to the walking group were given a training diary to record their walks and note the day, time of day and duration of the walk. Subjects were also required to rate their perceived exertion during the walk on the Borg 15-grade scale [20]. In addition, subjects noted their HR immediately following the walk, having previously been instructed in carotid and radial artery palpation. Nine of the walkers, who had difficulty with artery palpation, were given heart rate monitors to monitor their heart rate. Each of these subjects were trained in the use of the monitor and given written instructions. All subjects were instructed regarding the importance of maintaining their usual activity and dietary habits throughout the study.

### Step counts

Twelve walkers and all of the controls wore a pedometer during weeks 0, 4 and 8 of the intervention (Yamax SW-200, Yamasa Corporation; Tokyo, Japan). They were instructed to wear the pedometer during all waking hours and record the number of steps taken at the end of each day. In addition, during weeks 4 and 8, those assigned to the walking group recorded their step count before and after each walking session in their training diary. Each subject was trained in the correct use of the pedometer and given written guidelines to follow.

### Statistical analysis

Physiological differences between groups at baseline were compared using independent *t*-tests. Non-parametric Kruskall-Wallis tests were used to compare the step counts of groups at baseline. Non-parametric Wilcoxon tests were used to compare paired step count data of the walking group (Walk-days vs. Rest-days). Physiological data were analysed using a 2-way ANOVA with repeated measures, with one factor between subjects (walkers vs. controls) and one factor within subjects (pre- vs. post-intervention).

## Results

Four individuals dropped out of the study due to: illness (1 control), moving job (1 control), family circumstances (1 walker) and lack of interest (1 walker). Due to equipment error at the end of the walking intervention, body fat measurement was only possible in 12 walkers and 12 controls. Due to problems in blood sampling we were only able to determine blood lipids (TC, HDL-C, LDL-C, TG) in 11 walkers and 9 controls. In addition one control subject, who had CRP values > 10 mg· L^-1^, was excluded from the analysis as CRP values of this order may be indicative of infection or trauma (Ridker, 2003). CRP data is therefore presented for 11 walkers and 8 controls. For all other parameters measured data are presented for the 21 walkers and 12 controls who completed the study. Physiological characteristics of the participants at baseline are shown in Table 1. There were no significant differences between groups at baseline for any variable (*P *> 0.05).

During week 0 (i.e. the week prior to commencing the intervention) daily step counts for the walking and control groups averaged 6437 ± 2285 and 6831 ± 2727 respectively. There was no significant difference in the week 0 step counts between groups (*P *> 0.05). Daily step counts for the walking group on days when prescribed walking was performed (Walk-days), on days when no prescribed walking was performed (Rest-days) and all days during the programme are shown in Table 2. Walkers took significantly more steps on Walk-days compared to Rest-days (*P *< 0.001). Walkers undertook more voluntary steps (steps per day not including any accrued from prescribed walking) on Rest-days (5803 ± 2749) than on Walk-days (4567 ± 2639) (*P *< 0.05). During the intervention, mean step counts for the control group averaged 6470 ± 1709.

Subjects assigned to the walking group completed a 45 minute walk on two days of the week, at approximately 62.0 ± 7.1% predicted HR_max_. The walks elicited a mean RPE of 12.6 ± 0.9 and consisted of 4736.4 ± 539.2 steps. Subjects completed 83.9 ± 18.9% of prescribed sessions.

Table 3 shows measurements made at baseline and post-intervention. There were significant differences in the change in systolic BP and body fat percentage between groups from pre- to post-intervention as identified by the group-by-time interaction (*P *< 0.05). Systolic BP for the walking group decreased from 120.4 ± 19.7 mm Hg at baseline to 115.4 ± 17.7 mm Hg at post intervention. Body fat percentage of the walking group was 28.0 ± 5.8 and 27.9 ± 5.6 at pre- and post-intervention respectively. No significant changes were observed in body mass, waist and hip circumference, diastolic BP or lipid variables.

Mean values for CRP in the walking group at pre- and post-intervention were 1.9 ± 1.7 and 1.6 ± 1.5 mg· L^-1 ^respectively (n = 11). Corresponding values for controls were 1.5 ± 1.5 and 1.5 ± 1.3 mg· L^-1 ^at pre- and post-intervention respectively (n = 8). There were no significant effects observed. One subject, who had CRP values > 10 mg· L^-1^, was excluded from the analysis as CRP values of this order may be indicative of infection or trauma (Ridker, 2003).

Mean HR for the walking group during the treadmill test was 116.5 ± 8.5 beats· min^-1 ^and 112.0 ± 9.3 beats· min^-1 ^at pre- and post-intervention respectively. Corresponding values for the control group were 121.2 ± 14.7 and 118.1 ± 13.0 beats· min^-1 ^at pre- and post-intervention respectively. Blood lactate values for walkers at pre-and post-intervention were 1.6 ± 0.5 and 1.5 ± 0.5 mmol· L^-1 ^respectively. Pre- and post-intervention values for the control group were 1.5 ± 0.5 and 1.7 ± 0.5 mmol· L^-1^. Following training RPE during the treadmill test reduced from 11.2 ± 1.4 to 10.8 ± 1.4 in the walking group. A similar reduction was observed in controls, who demonstrated RPE values of 11.3 ± 2.1 and 10.8 ± 1.8 at pre- and post-intervention respectively. No significant differences were observed between groups in HR, blood lactate or RPE.

## Discussion

The main finding of the present study is that 45 minutes self-paced walking, two days per week, decreases systolic BP, but has no discernable effects on fitness, body mass, waist/hip circumferences, diastolic BP, CRP or lipoproteins, in previously sedentary employees. The novel aspect of this investigation is that it is the first to use a low frequency walking programme with adults < 60 years of age, and the first to examine the effects of walking, without dietary intervention, on CRP levels. The study augments the limited body of evidence on the efficacy of worksite physical activity interventions.

A significant effect was observed between groups for the change in systolic BP from pre- to post-intervention. Systolic BP was reduced by 5.0 ± 7.9 mm Hg in the walking group following eight weeks of training. A reduction of this magnitude is in general agreement with the findings of a meta-analysis on the effect of aerobic exercise on BP in normotensive adults [21]. In such individuals a modest reduction in BP confers prophylaxis against CVD morbidity and mortality as CVD risk is manifest on an approximately linear continuum with BP [22]. The benefit of such a reduction is supported by cross-sectional evidence showing that individuals in the normal range (120–128 systolic BP, and 81–84 diastolic BP) have almost a three-fold increase in risk over subjects with lower baseline levels [23]. Reductions in BP of this order on a population level may offer significant public health gains. A downward shift of about 2 mm Hg in the BP distribution of the general population should result in an annual reduction in stroke, coronary heart disease and all-cause mortality of about 6%, 4%, and 3% respectively [24].

Few other studies [25,26] have investigated the BP-lowering effects of walking in adults with mean baseline SBP values of less than 130 mm Hg. The present study is the first to show positive changes in SBP in normotensive adults following regular walking on just two days per week. Given the findings of the present study, the health benefits of lowered blood pressure, and the fact that walking is the most popular form of physical activity in the European Union [27], it is reasonable to suggest that continued and increased emphasis should be placed on increasing the proportion of adults who participate in this form of activity [21].

A significant effect was observed between groups for change in body fat percentage from pre- to post-intervention. Relative to an increase in controls, the body fat percentage of walkers remained stable. The increases in body fat percentage of the control group may, however, be the result of over-cautious control subjects. It is possible that a desire 'not to be active' may have resulted in marginally lower than normal physical activity levels thus contributing to worsened adiposity.

Participants in the present study are classified as being at moderate risk of future cardiovascular events according to their baseline CRP levels [28]. In the present investigation, although not statistically significant, CRP decreased by 15.8% following training in walkers, compared to 6.7% in controls. Furthermore, CRP values tended to decrease more notably in those with higher baseline values. Several cross-sectional studies have observed an inverse association between physical activity and CRP levels [29-33]. However, intervention studies examining the effects of training on CRP levels are less plentiful. There are only four exercise training interventions identified in the published literature [34-37], which reported reductions in CRP following training of 17, 35, 31 and 45 % respectively. Although the magnitude of CRP reduction observed in the present study (15.8%) is less than reported in the other exercise interventions, the exercise prescribed in the present study was considerably less (90 min· wk^-1 ^vs. 150–420 min· wk^-1^). Given the varied training regimes in the aforementioned studies, and the paucity of data in the area, comparisons are difficult to draw. Further research is required to elucidate the relationship between exercise levels (frequency, intensity, volume) and changes in CRP, and the effects of training in various population groups. The present study augments the very limited body of evidence on the effects of training on resting CRP concentrations.

Heart rate and blood lactate responses to submaximal workloads were used in the present study to monitor changes in aerobic fitness. No significant effects were observed for changes in heart rate or lactate response to submaximal workloads. The authors know of one other study in the published literature that has used the same walking prescription as the present investigation. Eighty women, aged 60–70 years progressed to walking 45 minutes 2 d· wk^-1 ^[10]. Following 26 weeks of training at approximately 67 – 78% HR_max_, heart rate during a submaximal exercise test was reduced. Failure to evoke changes in aerobic fitness in the present study, despite the same volume of exercise, may be due to differences in training intensity (67–78% HR_max _vs. ~ 62% HR_max_) or differences in the length of the intervention (26 weeks vs. 8 weeks) [38]. It has been postulated that the minimal intensity for improving cardiorespiratory fitness is 30% maximal oxygen uptake reserve/~60% HR_max _for lower-fit subjects [39]. The fact that the subjects in the present study exercised extremely close to the minimum threshold, or that they exercised for only eight weeks, could explain the lack of change in indices of aerobic fitness.

A systematic review of 32 cross-sectional, prospective observational and intervention studies, suggested that for adults, 7000 – 13,000 steps per day can be expected [40]. Baseline daily step counts from the present study are below this range (steps for the walking and control groups averaged 6437 ± 2285 and 6831 ± 2727 respectively), suggesting a comparatively sedentary sample for this age group. During the intervention, walkers significantly increased the total number of steps taken on Walk-days compared to Rest-days, indicating greater amounts of activity as a result of the walking programme. However, in the present study, there was a significant difference between the number of voluntary steps taken on Walk-days compared to the number of steps taken on Rest-days. This suggests that employees decreased their non-programme activity during Walk-days. A compensatory decline in both energy expenditure and steps in physical activity during the reminder of the day, in individuals who begin an exercise programme has been reported previously [41,42]. This may be due to a decrease in spontaneous physical activity and/or a reduction in voluntary physical activities [41].

The walking intervention was well tolerated by the employees in the present study. No injuries were reported during the programme, most likely due to the mode of exercise and that fact that low injury rates are associated with moderate intensity activity [43]. This finding is important because fear of sustaining an injury, and stopping activity because of an injury, have both been associated with failure to start or maintain a physically active lifestyle [44] Compliance was high with walkers completing 83.9 ± 18.9% of prescribed sessions, while attrition was low with only two walkers and two controls dropping out of the study. In addition, only one subject cited lack of interest as the reason for discontinued participation. Allowing people to exercise at their own pace may enhance comfort and enjoyment during exercise and may have contributed to the high compliance and low attrition levels in the present study. Additionally, existing channels of communication and opportunities for support may contribute to the use of the workplace as a positive setting for the promotion of physical activity [45]. These characteristics should be taken into account when public health professionals are designing programmes and prescribing physical activity within the workplace.

The limitations of this study need to be considered when interpreting findings. The CRP assay used in the present study did not detect CRP in the blood samples of 6 subject (3 walkers 3 controls) as they were below the detectable level of < 0.5 mg· L^-1^. However, apart from one subject, the same individuals were below detectable limits at both sampling times, thus limiting the resulting effect on observed changes over time.

## Conclusion

In summary, the findings of the present study suggest that self-paced walking 45 min, 2 d· wk^-1 ^for eight weeks at ~ 62% HR_max_, reduces systolic BP and prevents an increase in body fat, in previously sedentary employees. This walking prescription, however, failed to induce significant improvements in fitness, diastolic BP, body mass, serum lipids and CRP levels. The walking programme was associated with high adherence. These findings support the use of a twice-weekly, self-paced, worksite based, walking programme, to improve activity levels and systolic BP in previously sedentary employees. There is little evidence however to support the use of this exercise prescription for improvements in other markers of CVD risk. This walking prescription may therefore be useful as a stepping-stone to further increase levels of exercise, which may then provide greater benefits.

## Competing interests

The author(s) declare that they have no competing interests.

## Authors' contributions

MHM, EMM and CAGB were responsible for the design of the study

EMM was responsible for data collection

LGH was responsible for blood profiling

AMN was responsible for statistical analysis

MHM and EMM were responsible for preparing the manuscript

All authors read and approved the final manuscript

## Pre-publication history

The pre-publication history for this paper can be accessed here:



## Figures and Tables

**Table 1:** Physiological characteristics of subjects at baseline.

Variable	**Walking group**	**Control group**
n	21	12
Age (yr)	41.4 (7.5)	40.8 (10.0)
Height (cm)	166.5 (10.3)	167.1 (11.9)
Mass (kg)	75.3 (21.1)	68.4 (14.9)
BMI (kg· m^-2^)	26.8 (5.6)	24.4 (3.6)
Body fat (%)	28.0 (5.8)	25.9 (8.3)
Systolic BP (mm Hg)	120.4 (19.7)	116.5 (13.0)
Diastolic BP (mm Hg)	77.2 (9.4)	74.6 (9.0)
Waist circumference (cm)	84.8 (13.9)	83.2 (13.8)
Hip circumference (cm)	101.4 (9.0)	100.4 (6.7)
Total cholesterol (mmol· L^-1^)	5.7 (1.1)	5.7 (1.3)

Values are mean (SD)

Body fat percentages are based upon data from 12 walkers and 12 controls.

Blood lipids (TC, HDL-C, LDL-C, TG) are based upon data from 11 walkers and 9 controls.

CRP levels are based upon data from 11 walkers and 8 controls

All other variables are based upon data from 21 walkers and 12 controls.

**Table 2:** Daily step counts for the walking group (n = 12)

	Mean	**SD**	Range
Walk days	9303*	2665	6220 – 16392
Rest days	5803*	2749	3365 – 11730
All days	6801	2626	4180 – 13062

Values significantly different (*P *< 0.001)

Walk days, days when prescribed walking was performed; Rest days, days when prescribed walking was not performed.

**Table 3:** Anthropometric measures, blood pressure, lipid and C-Reactive Protein variables for Walkers and Controls at pre- and post-intervention.

**Variable**	**Walkers**		**Controls**	
	**Pre**	**Post**	**Pre**	**Post**

Body mass (kg)	75.3 (21.1)	75.7 (22.1)	68.4 (14.9)	69.6 (15.1)
Body fat (%)	28.0 (5.8)	27.9 (5.6) *	25.9 (8.3)	27.7 (7.8) *
Waist girth (cm)	84.8 (13.9)	84.0 (14.4)	83.2 (13.8)	83.4 (13.9)
Hip girth (cm)	101.4 (9.0)	101.0 (8.9)	100.4 (6.7)	100.7 (6.9)
Systolic BP (mm Hg)	120.4 (19.7)	115.4 (17.7) *	116.5 (13.0)	118.5 (9.8) *
Diastolic BP (mm Hg)	77.2 (9.4)	76.0 (10.2)	74.6 (9.0)	73.6 (6.6)
TC (mmol· L^-1^)	5.7 (1.1)	6.0 (1.3)	5.7 (1.3)	6.1 (1.7)
HDL-C (mmol· L^-1^)	1.1 (0.3)	1.3 (0.5)	1.1 (0.3)	1.4 (0.5)
LDL-C (mmol· L^-1^)	4.1 (1.1)	4.0 (1.4)	4.1 (1.2)	4.2 (1.6)
TG (mmol· L^-1^)	1.4 (1.2)	1.4 (1.1)	1.2 (0.6)	1.3 (0.6)
CRP (mg· L^-1^)	1.9 (1.7)	1.6 (1.5)	1.5 (1.5)	1.5 (1.3)

* Significant difference between groups from pre- to post-intervention. *P *< 0.05.

TC, total cholesterol; HDL-C, high density lipoprotein cholesterol; LDL-C, low density lipoprotein cholesterol; TG, triglycerides; CRP, C-Reactive Protein. Values are mean (SD).

Body fat percentages are based upon data from 12 walkers and 12 controls.

Blood lipids (TC, HDL-C, LDL-C, TG) are based upon data from 11 walkers and 9 controls.

CRP levels are based upon data from 11 walkers and 8 controls

All other variables are based upon data from 21 walkers and 12 controls.

## References

[B1] Health Promotion Agency (2001). A health economics model. The cost benefits of the Physical Activity Strategy for Northern Ireland - a summary of key findings.

[B2] Pate RR (1995). Physical activity and health: Dose-response issues. Res Q Exerc Sport.

[B3] Institute of Medicine of the National Academies (2002). Dietary Reference Intakes for Energy, Carbohydrate, Fiber, Fat, Fatty Acids, Cholesterol, Protein, and Amino Acids (Macronutrients).

[B4] Haapanen N, Miilnapalo S, Vuori I, Oja P, Pasanen M (1996). Characteristics of Leisure Time Physical Activity Associated with Decreased Risk of Premature All-Cause and Cardiovascular Disease Mortality in Middle-aged Men. American Journal of Epidemiolgy.

[B5] Pate RR, Pratt M, Blair SN, Haskell WL, Macera CA, Bouchard C, Buchner D, Ettinger W, Heath GW, King AC, Kriska A, Leon AS, Marcus BH, Morris J, Paffenbarger RS, Patrick K, Pollock ML, Rippe JM, Sallis J, Wilmore JH (1995). Physical-Activity and Public-Health - a Recommendation from the Centers-for-Disease-Control-and-Prevention and the American- College-of-Sports-Medicine. JAMA-J Am Med Assoc.

[B6] New Zealand National Health Committee (1998). Active For Life: a call for action. The health benefits of physical activity.

[B7] European Heart Network (1999). Physical Activity and Cardiovascular Disease Prevention in the European Union.

[B8] Davison R, Mutrie N, Nash AS, Kelly MPT, Dargie HJ (1992). Walk for health?. Journal of Sports Science.

[B9] Wenger HA, Bell GJ (1986). The Interactions of Intensity, Frequency and Duration of Exercise Training in Altering Cardiorespiratory Fitness. Sports Med.

[B10] Hamdorf PA (1992). Physical training effects on the fitness and habitual activity patterns of elderly women. Arch Phys Med Rehabil.

[B11] Hamdorf PA, Penhall RK (1999). Walking with its training effects on the fitness and activity patterns of 79-91 year old females. Aust N Z J Med.

[B12] Ridker PM, Rifai N, Rose L, Buring JE, Cook NR (2002). Comparison of C-reactive protein and low-density lipoprotein cholesterol levels in the prediction of first cardiovascular events. N Engl J Med.

[B13] Jones PRM, Hunt MJ, Brown TP, Norgan NG (1986). Waist-Hip Circumference Ratio and its relation to age and overweight in British men. Human Nutrition: Clinical Nutrition.

[B14] American College of Sports Medicine (2000). Guidelines for Exercise Testing and Prescription.

[B15] Friedewald WT, Levy RI, Fredrickson DS (1972). Estimation of the concentration of low-density lipoprotein cholesterol in plasma, without use of the preparative ultracentrifuge.. Clinical Chemistry.

[B16] Dill DB, Costill DL (1974). Calculation of percentage changes in volumes of blood, plasma, and red cells in dehydration. Journal of Applied Physiology.

[B17] Krummel D, Etherton TD, Peterson S, Krisetherton PM (1993). Effects of Exercise on Plasma-Lipids and Lipoproteins of Women. Proc Soc Exp Biol Med.

[B18] Thompson PD, Crouse SF, Goodpaster B, Kelley D, Moyna N, Pescatello L (2001). The acute versus the chronic response to exercise. Medicine and Science in Sports and Exercise.

[B19] Murtagh EM, Boreham CAG, Murphy MH (2002). Speed and exercise intensity of recreational walkers. Prev Med.

[B20] Borg GAV (1982). Psychophysical Bases of Perceived Exertion. Med Sci Sports Exerc.

[B21] Kelley GA, Kelley KS, Tran ZV (2001). Walking and resting blood pressure in adults: a meta-analysis. Prev Med.

[B22] Lever AF, Boushel R, Saltin BBRSNMJ (2000). Hypertension. Exercise and Circulation in Health and Disease.

[B23] Blair SN, Goodyear NN, Gibbons LW, Cooper KH (1984). Physical-fitness and incidence of hypertension in healthy normotensive men and women.. Journal of the American Medical Association.

[B24] Whelton PK (1994). Epidemiology of hypertension. Lancet.

[B25] Kingwell BA, Jennings GL (1993). Effects of Walking and Other Exercise Programs Upon Blood- Pressure in Normal Subjects. Med J Aust.

[B26] Braith RW, Pollock ML, Lowenthal DT, Graves JE, Limacher MC (1994). Moderate and High-Intensity Exercise Lowers Blood-Pressure in Normotensive Subjects 60 to 79 Years of Age. Am J Cardiol.

[B27] Vaz de Almeida MD, Graca P, Afonso C, D'Amicis A, Lappalainen R, Damkjaer S (1999). Physical activity levels and body weight in a nationally representative sample in the European Union. Public Health Nutrition.

[B28] Ridker PM (2003). Clinical Application of C-Reactive Protein for Cardiovascular Disease Detection and Prevention. Circulation.

[B29] Rohde LEP, Hennekens CH, Ridker PM (1999). Survey of C-reactive protein and cardiovascular risk factors in apparently healthy men. Am J Cardiol.

[B30] Geffken DF, Cushman M, Burke GL, Polak JF, Sakkinen PA, Tracy RP (2001). Association between physical activity and markers of inflammation in a healthy elderly population. Am J Epidemiol.

[B31] Abramson JL, Vaccarino V (2002). Relationship between physical activity and inflammation among apparently healthy middle-aged and older US adults. Arch Intern Med.

[B32] Ford ES (2002). Does exercise reduce inflammation? Physical activity and C- reactive protein among US adults. Epidemiology.

[B33] Wannamethee SG, Lowe GDO, Whincup PH, Rumley A, Walker M, Lennon L (2002). Physical activity and hemostatic and inflammatory variables in elderly men. Circulation.

[B34] Tisi PV, Hulse M, Chulakadabba A, Gosling P, Shearman CP (1997). Exercise training for intermittent claudication: Does it adversely affect biochemical markers of the exercise-induced inflammatory response?. European Journal of Vascular and Endovascular Surgery,.

[B35] Smith JK, Dykes R, Douglas JE, Krishnaswamy G, Berk S (1999). Long-term exercise and atherogenic activity of blood mononuclear cells in persons at risk of developing ischemic heart disease. JAMA-J Am Med Assoc.

[B36] Mattusch F, Dufaux B, Heine O, Mertens I, Rost R (2000). Reduction of the plasma concentration of C-reactive protein following nine months of endurance training. Int J Sports Med.

[B37] Roberts CK, Wegge JK, Ngo TH, Barnard RJ (2003). Effect of diet and exercise intervention on inflammation and adhesion molecules in postmenopausal women. Medicine and Science in Sports and Exercise.

[B38] Pollock ML, Gaesser GA, Butcher JD, Despres JP, Dishman RK, Franklin BA, Garber CE (1998). The recommended quantity and quality of exercise for developing and maintaining cardiorespiratory and muscular fitness, and flexibility in healthy adults. Med Sci Sports Exerc.

[B39] Swain DP, Franklin BA (2002). VO2 reserve and the minimal intensity for improving cardiorespiratory fitness. Medicine and Science in Sports and Exercise,.

[B40] Tudor-Locke CE, Myers AM (2001). Challenges and Opportunities for Measuring Physcial Activity in Sedentary Adults. Sports Medicine.

[B41] Goran MI, Poehlman ET (1992). Endurance training does not enhance total energy-expenditure in healthy elderly persons. American Journal of Physiology,.

[B42] Hagiwara A, Hayashi Y, Nakamura Y, Muraoka I (2000). Effects of group- versus home-based walking intervention on lifestyle activity. Jpn J Phys Fit Sports Med.

[B43] Franklin BA, Dishman RK (1988). Program factors that influence exercise adherence: Practical adherence skills for clinical staff.. Exercise Adherence: Its impact on Public Health.

[B44] Hardman AE, Stensel DJ (2003). Physical Activity and Health..

[B45] Jaffee L, Lutter JM, Rex J, Hawkes C, Bucaccio P (1999). Incentives and barriers to physical activity for working women.. American Journal of Health Promotion,.

